# TALE-directed local modulation of H3K9 methylation shapes exon recognition

**DOI:** 10.1038/srep29961

**Published:** 2016-07-21

**Authors:** Nicole I. Bieberstein, Eva Kozáková, Martina Huranová, Prasoon K. Thakur, Zuzana Krchňáková, Michaela Krausová, Fernando Carrillo Oesterreich, David Staněk

**Affiliations:** 1 Institute of Molecular Genetics of the ASCR, v. v. i., Vídeňská 1083, 142 20 Prague 4, Czech Republic; 2 Max Planck Institute of Molecular Cell Biology and Genetics, 01307 Dresden, Germany

## Abstract

In search for the function of local chromatin environment on pre-mRNA processing we established a new tool, which allows for the modification of chromatin using a targeted approach. Using Transcription Activator-Like Effector domains fused to histone modifying enzymes (TALE-HME), we show locally restricted alteration of histone methylation modulates the splicing of target exons. We provide evidence that a local increase in H3K9 di- and trimethylation promotes inclusion of the target alternative exon, while demethylation by JMJD2D leads to exon skipping. We further demonstrate that H3K9me3 is localized on internal exons genome-wide suggesting a general role in splicing. Consistently, targeting of the H3K9 demethylase to a weak constitutive exon reduced co-transcriptional splicing. Together our data show H3K9 methylation within the gene body is a factor influencing recognition of both constitutive and alternative exons.

The removal of introns and ligation of exons in the process of pre-mRNA splicing takes place predominantly co-transcriptionally^1^,^2^,^3^. In budding yeast, an RNA polymerase II (Pol II) pause site in the terminal exon ensures that splicing is completed before termination of transcription, suggesting that co-transcriptional splicing is of functional importance^2^,^4^. In fact, mounting evidence suggests transcription, chromatin and pre-mRNA splicing are functionally coupled. The first evidence implying chromatin affects alternative splicing came from genome-wide ChIP-Seq studies that correlated the presence of certain histone modifications with exon inclusion rates^5^,^6^. However, determining whether these histone marks are causative for splicing changes or simply a consequence of splicing cannot be addressed by this correlative approach. While it is widely assumed that chromatin modifications modulate alternative splicing, several studies have provided evidence that H3K4 and H3K36 methylation is splicing dependent^7^,^8^,^9^. These data suggest a more complex regulatory network where splicing can feed back on chromatin to label histones for the following rounds of transcription.

In order to experimentally determine the role of chromatin in alternative splicing regulation, most studies have used a global approach to perturb histone modifications genome-wide, such as small molecule inhibitors or knockdown/overexpression of histone modifying enzymes (HMEs)^10^,^11^,^12^,^13^,^14^,^15^. As this method globally affects the transcriptional program of the cell, secondary effects cannot be fully excluded. The central question remains, what is the minimal amount of chromatin change required to influence splicing and how local is this effect? In this study, we set out to determine the role of chromatin by direct, local manipulation of the chromatin environment at a selected target exon. We use *Transcription-Activator-Like-Effector* (TALE) domains to tether HMEs to a target exon locus, analogous to TALENs for genome engineering or TALE-TFs for transcriptional regulation^16^,^17^,^18^. This strategy has been successfully utilized to modify chromatin modifications at enhancers^19^. As proof of principle, we chose to investigate the effect of H3K9 and H3K36 methylation^11^,^12^,^15^,^20^,^21^, histone marks previously shown to modulate alternative splicing, on alternative splicing of the endogenous human fibronectin EDB exon, a widely studied example of a cassette exon^22^,^23^,^24^. We further studied the global distribution of H3K9me3 within genes and showed this histone mark plays an unanticipated role in splicing of constitutive exons.

## Results

### TALEs for targeted modulation of chromatin

**Figure 1:**
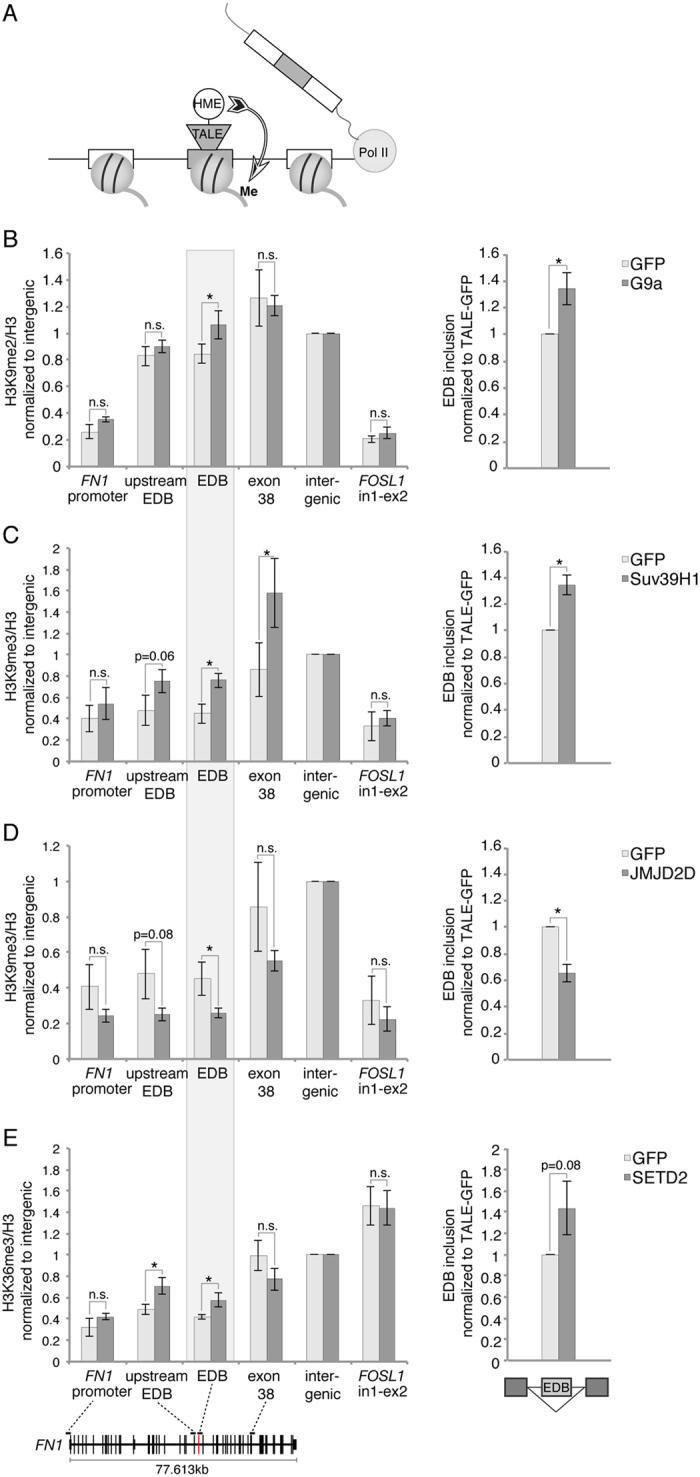
Local manipulation of histone modifications by TALE-HME affects alternative splicing of the target exon. (**A**) Schematic representation of the approach. A TALE domain was assembled to bind the target exon and fused to HMEs. Upon binding of TALE-HME to the target exon, the local chromatin environment is modified. (**B–E**) Upon transient transfection with TALE-HME or TALE-GFP as a control, the effect on chromatin was monitored by ChIP (left panel) and EDB inclusion levels were assessed by RT-qPCR (right panel). ChIP signals are calculated as immunoprecipitated DNA over input signal and normalized to total H3 and an intergenic region on chromosome 10. Amplicon positions for FN1 are indicated in the gene diagram at the bottom. EDB inclusion rates are calculated as the ratio of the EDB exon to the upstream constitutive exon 24 and normalized to TALE-GFP. Mean ± SEM are shown, n = 3–4. Statistical significance of all results was analyzed by t-test and significant changes (p < 0.05) were indicated by * .

To test whether locally restricted changes in the chromatin context can directly affect splicing of an alternative exon, we altered H3K36 and H3K9 methylation at the EDB exon in human fibronectin (*FN1*). Methylation of H3K9 and H3K36 were previously proposed to regulate alternative splicing^11^,^12^,^15^,^20^,^21^, and splicing of the EDB exon was described to be sensitive to the chromatin environment^13^,^23^,^24^. To specifically and locally target chromatin around the EDB exon we designed a TALE domain recognizing 23 nt of the plus strand close to the 3′ splice site (SS) of the EDB exon using the TAL Effector Targeter^25^ (https://tale-nt.cac.cornell.edu/node/add/single-tale). No off targets in the human genome were predicted for this binding sequence. To modulate H3K9 methylation, the TALE domain was fused to the H3K9 tri-methyltransferase Suv39H1, the catalytic domain of the di-methyltransferase G9a (EHMT2) or the H3K9me2/3 demethylase JMJD2D (KDM4D)^26^,^27^,^28^,^29^. For H3K36me3 we selected the methyltransferase ASH1L and the catalytically active domain of SETD2^30^,^31^ (Fig. 1A). The HA-tag was included as a linker between the TALE domain and HME to allow detection of fusion proteins by Western blot (Supplementary Fig. S1). As a control, the TALE domain was fused to GFP and all results were normalized or compared to the TALE-GFP construct in order to exclude unspecific effects due to the binding of the TALE construct to DNA.

### Local changes in chromatin alter EDB inclusion

TALE-HME or TALE-GFP constructs were transiently transfected into HeLa cells and the effect of TALE-HME expression on chromatin was monitored by chromatin immunoprecipitation (ChIP). Tethering of the H3K9me2 methyltransferase construct TALE-G9a to the EDB exon resulted in a significant ~20% increase in H3K9me2 at the target site, while the immediate upstream exon (1.5 kb distant from EDB), a far downstream exon (17.8 kb distant from EDB) and the promoter of *FN1* were unaffected (Fig. 1B, left panel). Likewise, methylation of a non-targeted gene, *FOSL1*, remained unchanged. The effect of chromatin alteration on alternative splicing was assessed using RT-qPCR. Expression of TALE-G9a resulted in a ~30% increase in EDB inclusion compared to the tethering of control TALE-GFP, thus proving the principle that a local change in the chromatin environment impacts alternative splicing (Fig. 1B, right panel).

Similarly to H3K9me2, targeting the H3K9me3 methyltransferase Suv39H1 led to higher levels of H3K9me3 at the target exon (Fig. 1C). However, here methylation was also elevated at both the downstream exon and the immediate upstream exon, suggesting the possibility of H3K9me3 spreading, as previously described for heterochromatin^32^. Nevertheless, the promoter region of *FN1* and the control gene *FOSL1* were not affected indicating gene specific targeting of Suv39H1. Analogous to H3K9 dimethylation by TALE-G9a, increased H3K9 trimethylation promoted EDB inclusion. Since H3K9me2 serves as a substrate for Suv39H1, it is possible the increased H3K9 dimethylation upon TALE-G9a is converted to H3K9me3 by endogenous enzymes. However, TALE-G9a tethering did not exhibit elevated H3K9me3 levels along *FN1*, indicating that H3K9me2 and H3K9me3 can independently promote exon inclusion (Supplementary Fig. S1). To further test the role of H3K9me2/3 on alternative splicing, the H3K9 demethylase JMJD2D was targeted to the EDB exon subsequently leading to reduced H3K9me3 at the target site, which in turn enhanced exon skipping (Fig. 1D). These results demonstrate the local level of H3K9 methylation directly impacts splicing of the EDB exon and that a higher methylation promotes exon inclusion.

Next, we tested the effect of two H3K36me3 methyltransferases - SETD2 and ASH1L. TALE-SETD2 locally increased H3K36me3 levels at the target exon and the immediate upstream exon but the changes in alternative splicing were below statistical significance (Fig. 1E). TALE-ASH1L had no effect on the chromatin level nor on alternative splicing (Supplementary Fig. S1). However, the latest construct was poorly expressed (Supplementary Fig. S1).

### Internal exons are tri-methylated at H3K9

**Figure 2:**
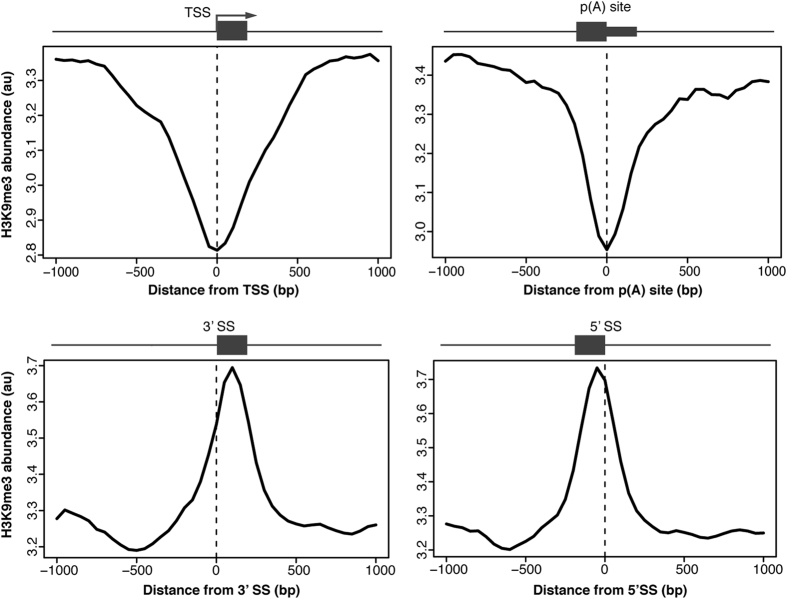
H3K9me3 signal at internal exons. Relative H3K9me3 abundance profiles in HeLa cells 1kb up and downstream of transcriptional start sites (TSS, upper left panel), polyadenylation sites (p(A) site, upper right panel), 3′ splice sites (lower left panel) and 5′ splice sites (lower right panel) of internal exons were plotted versus relative genomic positions 1 kb up and downstream of gene architecture hallmark of interest. Genome wide abundance profiles were derived from chromatin immunoprecipitation sequencing (ChIP-seq) experiments performed in the ENCODE project. Schematic gene architecture hallmarks are displayed above. See also Supplementary Fig. S2 for K562 and MCF-7 analysis.

Given the positive effect of local H3K9me3 on alternative exon inclusion (Fig. 1^21^,^33^), we speculated this histone modification may help to recognize exons in general. We first determined H3K9me3 abundance around gene architecture landmarks using publicly available H3K9me3 ChIP-seq data from three different human cell lines (K562, HeLa and MCF-7) generated by the ENCODE project^34^. H3K9me3 was depleted around transcriptional start sites and poly(A) sites and enriched around internal exons in all tested cells (Fig. 2 and Supplementary Fig. S2). While H3K9 profiles at internal exons resemble the general distribution of nucleosomes^5^,^6^, it confirms that nucleosomes localized on internal exons carry H3K9 trimethylation and is consistent with the model that this histone modification is important for exon recognition.

### H3K9me3 influences co-transcriptional splicing of constitutive exons

**Figure 3:**
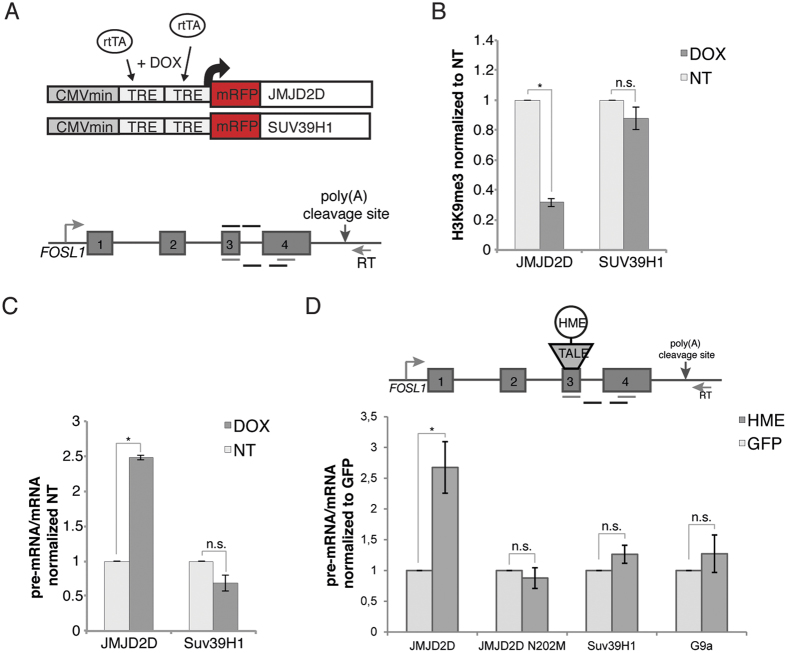
H3K9me3 promotes co-transcriptional splicing of *FOSL1*. (**A**) Stable U2OS-TO cell lines for doxycycline inducible overexpression of JMJD2D and Suv39H1 were established to test the effect of H3K9me3 on co-transcriptional splicing of exons 3 to 4 of *FOSL1*. Schematic representation of the Tet-On constructs and the endogenous *FOSL1* gene are shown. To select nascent transcripts a primer downstream of the poly(A) cleavage site was used for reverse transcription. qPCR primers used for detection of pre-mRNA or mRNA are indicated below the gene diagram; above are the primers for ChIP. (**B**) H3K9me3 ChIP on FOSL1 exon 3 showing reduced H3K9me3 levels after JMJD2D overexpression, while Suv39H1 had no additional effect. ChIP signals are calculated as immunoprecipitated DNA over input and normalized to the uninduced control cells. Mean ± SEM are shown, n = 3. Statistical significance of all results was analyzed by t-test and significant changes (p < 0.05) were indicated by*. (**C**) Unspliced pre-mRNA accumulates after JMJD2D overexpression and depletion of H3K9me3 levels, while Suv39H1 had no effect on co-transcriptional splicing of FOSL1 exons 3–4 as determined by RT-qPCR. Ratios of unspliced pre-mRNA to spliced mRNA are normalized to uninduced control cells. Mean ± SEM are shown, n = 3. Statistical significance of all results was analyzed by t-test and significant changes (p < 0.05) were indicated by*. (**D**) Co-transcriptional splicing of FOSL1 exons 3–4 was assessed after targeting H3K9 methyltransferases and demethylases by TALE-HMEs binding exon 3. A primer downstream of the poly(A) cleavage site was used for reverse transcription to select nascent transcripts. Tethering of JMJD2D, but not the catalytically inactive JMJD2D-N202M, resulted in accumulation of unspliced transcripts. In contrast, tethering of methyltransferases G9a or Suv39H1 did not further improve co-transcriptional splicing. Ratios of unspliced pre-mRNA to spliced mRNA are normalized to TALE-GFP. Mean ± SEM are shown, n = 3. Statistical significance of all results was analyzed by t-test and significant changes (p < 0.05) were indicated by*.

To experimentally test the effect of H3K9 methylation on splicing efficiency of constitutive exons we analyzed co-transcriptional splicing of the exon 3 in *FOSL1*. This exon is surrounded by weak splice sites and H3K9me3 might promote its inclusion similarly to the alternative EDB exon. To modulate H3K9me3 we utilized two alternative approaches - global overexpression of the methyltransferase Suv39H1 and demethylase JMJD2D and local targeting of HMEs via TALE to *FOSL1* exon 3. To avoid potential artifacts connected to the cell adapting to HME overexpression, we established stable cell lines containing Suv39H1 or JMJD2D under doxycycline inducible promoter. Suv39H1 or JMJD2D were tagged with mRFP to monitor their expression. Suv39H1-mRFP or JMJD2D-mRFP induction with doxycycline led to a global increase or decrease of H3K9me3, respectively (Supplementary Fig. 3). H3K9me3 ChIP on *FOSL1* exon 3 confirmed that induction of JMJD2D expression reduced H3K9me3 levels by 70%. However, overexpression of Suv39H1 had no effect on H3K9me3 levels (Fig. 3B). Since the H3K9me3 signal was already high in uninduced cells, further increase in methylation may not have been possible. To monitor the splicing efficiency of the constitutive exon 3 in *FOSL1*, we analyzed co-transcriptional splicing of nascent RNA to avoid potential artifacts due to high amount of spliced mRNA present in total RNA samples. We used a primer downstream of the poly(A) cleavage site for reverse transcription, thereby selecting for nascent transcripts still attached to chromatin through the transcription machinery^2^,^35^ (Fig. 3A). The ratio of pre-mRNA to mRNA revealed an accumulation of unspliced transcripts after JMJD2D overexpression and depletion of H3K9me3 (Fig. 3C). Suv39H1 overexpression had no additional effect in enhancing the co-transcriptional splicing of the constitutive exon, which was consistent with ChIP data, when we observed no increase in H3K9me3.

To further investigate the role of H3K9me3 in exon recognition, we utilized the TALE-HME system and expressed Suv39H1, G9a or JMJD2D fused with the TALE domain targeting *FOSL1* exon 3 (Supplementary Fig. 3). Consistent with global overexpression, tethering of JMJD2D significantly reduced splicing efficiency, while TALE-Suv39H1 or TALE-G9a had no effect (Fig. 3D). These results demonstrate H3K9me3 has a functional role in the splicing of constitutive exons.

## Discussion

In recent years there has been mounting evidence that pre-mRNA splicing is closely coupled with RNA synthesis and the chromatin environment of template DNA^4^,^36^. The original findings suggested that chromatin modifications modulate alternative splicing^11^,^13^,^14^,^21^,^23^,^37^,^38^ and further studies have shown that pre-mRNA splicing reciprocally feeds back on histone modifications^7^,^8^,^9^. Most studies have relied on global depletion/inhibition or overexpression of chromatin modifying enzymes. These experiments, though thoroughly controlled, can never exclude the possibility that the observed changes in alternative splicing are caused by downstream secondary effects. The first attempt to investigate local effects of chromatin on splicing utilized siRNA-induced chromatin methylation^22^. However, the molecular mechanism of how siRNAs modulate chromatin modifications in mammalian cells is still not fully understood^39^ and the histone marks altered by this approach can not be controlled. A recent study utilized a zinc finger protein to tether the histone methyltransferase G9a to the promoter region of a target gene. This method increased H3K9me2 at both the promoter and the downstream region within the gene body, thus leaving the question of how local chromatin at the target exon affects splicing unaddressed^15^. To address whether a change of local histone methylation can modulate splicing, we used TALEs as a tool to navigate HMEs to a specific and unique sequence within the targeted gene. Given the specificity of TALE binding, secondary effects due to global changes in the transcriptional output can be eliminated, thus providing a tool to directly test the influence of local histone modifications on splicing.

First we targeted the alternative exon EDB in the *FN1* gene, which was previously shown to be sensitive to chromatin regulation^13^,^23^. Four out of five tested TALE-HME constructs exhibited the desired effect on chromatin. SETD2 targeting increased H3K36 trimethylation at the target exon but we did not detect any robust changes in alternative splicing. H3K36me3 was reported to impact alternative splicing by the recruitment of splicing regulatory factors^11^,^12^,^20^. However, this effect is apparently context dependent and H3K36me3 alone is not sufficient to change the fate of the alternative target exon.

In contrast to H3K36me3, alteration of H3K9 methylation status exhibited a clear effect on the EDB exon. Expression of TALE fused with methyltransferases G9a or Suv39H1 increased H3K9me2 and me3 respectively, which in both cases promoted inclusion of the alternative exon. Conversely, TALE targeting of histone demethylase JMJD2D reduced H3K9me3, which reduced EDB inclusion. Furthermore, we showed that H3K9me3 is present over internal exons genome-wide, suggesting this histone mark plays a general role in exon recognition. Although the genome-wide abundance profiles mirror nucleosome position as derived from MNase ChIP-Seq data^5^,^6^, our data supports the fact that these nucleosomes carry H3K9me3 and this histone modification is a functional element in splicing regulation. Indeed, overexpression or TALE targeting of histone demethylase JMJD2D to constitutively spliced exon 3 of the *FOSL1* gene reduced co-transcriptional splicing of this exon. Based on the bioinformatic analysis and the JMJD2D expression data we conclude that H3K9me3 at nucleosomes within the gene body affect exon recognition. Two mechanistic models can explain the effect of H3K9me3 on splicing. First, methylated H3K9 is bound by HP1γ, which in turn can recruit the splicing regulatory protein SRSF1^15^. Alternatively, H3K9 methylation might affect alternative splicing through kinetic coupling by slowing down Pol II elongation^21^,^22^,^37^. Nucleosomes positioned over exons were previously proposed to act as speed bumps for Pol II elongation and H3K9 methylation on these exon-centered nucleosomes may increase the effect on Pol II speed^40^. Furthermore, splicing of the EDB exon was shown to be sensitive to Pol II elongation rate^13^,^23^,^24^, rendering kinetic coupling the most likely mechanism by which TALE-directed H3K9 methylation changes EDB inclusion rates.

In general, our data point to H3K9 methylation as the mark important for proper splicing pattern. This supports a model in which histone modifications together with DNA sequence, transcription machinery and the spliceosome form a complex unit, acting in cooperation to produce functional mRNA.

## Material and Methods

### Cell culture and treatments

HeLa and U2OS-TO cells were cultured in high glucose (4.5 g/l) DMEM (Sigma) supplemented with 100 U/ml penicillin, 100 μg/ml streptomycin (Penicillin/Streptomycin, Gibco) and 10% (v/v) fetal bovine serum (FBS, Gibco) at 37 °C and 5% CO_2_. Expression of mRFP-JMJD2D and mRFP-Suv39H1 in U2OS-TO cells was induced with 15 or 30 μg/ml doxycycline for 20 h. *FOSL1* was induced by addition of 2 μg/ml ionomycin calcium salt (LifeTechnologies) for 30 min.

### Plasmids and stable cell lines

TALE-domains targeting the EDB exon in human *FN1* and exon 3 in human *FOSL1* were designed using TAL Effector Targeter^25^ (https://tale-nt.cac.cornell.edu/node/add/single-tale) and assembled into pTALEN^41^ (Addgene) using the Golden Gate TALEN and TAL Effector Kit 2.0 (Daniel Voytas, Addgene)^17^. GFP or histone modifying enzymes Suv39H1, ASH1L, JMJD2D and the catalytically active domains of G9a and SETD2 were cloned into the pTALEN^41^ backbone using NaeI and XbaI restriction sites, replacing the nuclease domain. The HA-tag was included as a linker between the TALE-domain and the HME. Plasmids were transiently transfected into HeLa or U2OS-TO cells using Lipofectamin LTX reagent (LifeTechnologies) according to manufacturer’s instructions and incubated for 48 h.

Suv39H1 and JMJD2D were cloned into mRFP-C3 (Clontech) with BamHI/EcoRI or BamHI/XhoI restriction sites, respectively. The resulting Suv39H1-mRFP or JMJD2D-mRFP constructs were subcloned into pcDNA4/TO (LifeTechnologies) with KpnI/BamHI or HindIII/BamHI restriction sites, respectively. Stable cell lines in U2OS-TO cells were established after transfection with Lipofectamin LTX (LifeTechnologies) and selection with 300 μg/ml Zeocin (LifeTechnologies). Single-cell-colonies were expanded, tested for doxycycline inducible expression of Suv39H1-mRFP or JMJD2D-mRFP and individual clones were selected for experiments. To generate catalytically inactive JMJD2D, we introduced the point mutation N202M, which disrupts the coordination of α-ketoglutarate within JMJD2D catalytic site^42^. Mutagenic primers were designed using NEBaseChanger tool (F: 5′- TACAGCATCAtgTACCTGCACCTTGG - 3′; R: 5′ - AAGGTCCATGTCCTCTGTATG - 3′) and verified by DNA sequencing.

### RNA isolation, reverse transcription and qPCR

Cells were grown to 90% confluency in a 6-well tissue culture plate (~10^6 ^cells). RNA was isolated using TRIZOL reagent (LifeTechnologies), precipitated with isopropanol, resuspended in nuclease free water and treated with Turbo DNase (Ambion) according to manufacturer’s protocol. Reverse transcription was performed with SuperScript III (LifeTechnologies) using 2 μg of RNA. Total RNA was reverse transcribed with random hexamer primers, while nascent RNA from *FOSL1* was selectively enriched using a primer downstream of the poly(A) cleavage site^2^,^35^. The cDNA was analyzed by quantitative PCR. A list of primers is provided in the supplemental material.

### Antibodies

The following antibodies were used for ChIP: rabbit α-H3K9me3 (Abcam ab8898), rabbit α-H3K36me3 (Abcam ab9050), mouse α-H3K9me2 (Millipore 05-1249), rabbit α-H3 (Abcam ab1719) and mouse α-IgG (Sigma I5381).

For Western blot the following antibodies were used: mouse α-GAPDH (Abcam ab9484), rabbit α-mRFP (Apronex), rat α-HA (Roche), mouse α-GFP (Santa Cruz sc-9996), rabbit α-H3K9me3 (Abcam ab8898), mouse α-tubulin kindly provided by Pavel Draber (Institute of Molecular Genetics of the ASCR, Prague, Czech Republic), goat α-mouse-HRP (Jackson ImmunoResearch), goat α-rabbit-HRP (Jackson ImmunoResearch), goat α-rat-HRP (Santa Cruz sc-2006).

### Protein extraction and Western Plot

Proteins were isolated from TRIZOL fractions at the same time as RNA, precipitated with isopropanol and resuspended in NEST-2 buffer (50 mM Tris-HCl pH 6.8, 20 mM EDTA, 5% (w/v) SDS). Proteins were resolved on a 10% SDS-PAGE, blotted onto a nitrocellulose membrane and detected using the antibodies indicated above and SuperSignal Pico West (Thermo Fisher Scientific).

### Native Chromatin Immunoprecipitation

Native ChIP assays were performed as previously described^13^. Cells were grown to 90% confluency in a 6-well plate, washed twice and scraped in PBS with proteinase inhibitor cocktail (Calbiochem). Four wells were combined for one ChIP experiment and the native ChIP assay was performed as previously described^13^. A list of primers is provided in the supplemental material. ChIP signals were calculated as IP over Input (2^(Ct(input)–Ct(IP)^) and normalized to a non-transcribed intergenic control region.

### Cross-linked Chromatin Immunoprecipitation

Cross-linked ChIP assays were performed as previously described^13^. Cells were grown to 90% confluency in a 6-well plated, crosslinked with 1% formaldehyde in PBS for 10 min and ChIP assays were performed as previously described^13^. ChIP signals were calculated as IP over Input (2^(Ct(input)–Ct(IP)^) and normalized to a non-transcribed intergenic control region.

### H3K9me3 ChIP profiles

H3K9me3 ChIP-seq experiments for different cell lines were performed by the ENCODE project^34^ and are publicly available (https://www.encodeproject.org/experiments/). The annotated references were obtained from GENCODE (Release 19)^43^. Processed data were obtained in bigwig format for the human genome version 19 (hg19) of the HeLa (ENCFF000BBL), K562 (ENCFF000BYX) and MCF-7 (ENCFF000VFL) cell lines. Mean abundance profiles in a window 1 kb up- and downstream for annotated transcriptional start sites (TSSs), polyA-sites as well as 5′ SSs and 3′ SSs of internal exons were determined using Sitepro of CEAS package^44^.

## Additional Information

**How to cite this article**: Bieberstein, N. I. *et al*. TALE-directed local modulation of H3K9 methylation shapes exon recognition. *Sci. Rep.* **6**, 29961; doi: 10.1038/srep29961 (2016).

## Supplementary Material

Supplementary Information
