# Supplementary material for: TALE-directed local modulation of H3K9 methylation shapes exon recognition
Source: Sci Rep. 2016 Jul 21;6:29961. doi: 10.1038/srep29961 (PMC4954949; doi:10.1038/srep29961)
Supplement: Supplementary Information [file srep29961-s1.pdf]

SUPPLEMENTARY INFORMATION

TALE-directed modulation of H3K9 methylation shapes exon recognition

Nicole I. Bieberstein, Eva Kozáková, Martina Huranová, Prasoon K. Thakur, Zuzana Krchňáková, Michaela Krausová, Fernando Carrillo Oesterreich and David Staněk

Supplementary list of primers

Primers for quantitative PCR – chromatin immunoprecipitation:

**Target**	**sequence**
FN1 promoter (-505 bp*)	5´- TTGATGACCGCAAAGGAAAC -3´
	5´- TCGCAGCGAACAAAAGAGAT -3´
FN1 exon 24	5´- GGAAGAAGTGGTCCATGCTG -3´
(upstream of EDB, 41196 bp)	5´- GGGACACTTTCCTTGTCATCC -3´
FN1 exon 25 (EDB, 42658 bp)	5´- AGGTGCCCCAACTCACTGACC -3´
	5´- TGCCGCAACTACTGTGATGCGGTA -3´
FN1 exon 38 (60499 bp)	5´- CACCCAATTCCTTGCTGGTA -3´
	5´- GGACCACTTCTCTGGGAGGA -3´
intergenic region	5´- GGCTAATCCTCTATGGGAGTCTGTC -3´
	5´- CCAGGTGCTCAAGGTCAACATC -3'q
FOSL1 intron 1 – exon 2	5‘ – ACTGCCAAGCTGTGCTCTTT – 3‘
	5‘ – ACTGCCACTCATGGTGTTGA – 3‘
FOSL1 intron 3 – exon 4	5‘ – CCTCAGAACCCTGAGTCCAA – 3‘
	5‘ – CTTCTGCTTCTGCAGCTCCT – 3‘

Primers for quantitative PCR – Alternative splicing:

FN1 exon 24	5´- GGAAGAAGTGGTCCATGCTG-3´
(upstream EDB, 41196 bp)	5´- GGGACACTTTCCTTGTCATCC -3´
FN1 exon 25 (EDB, 42658 bp)	5´- AGGTGCCCCAACTCACTGACC -3´
	5´- TGCCGCAACTACTGTGATGCGGTA -3´

Primers for quantitative PCR – Co-transcriptional splicing:

RT primer downstream of poly(A)	5‘ – GGGACCTAGGGCTCCAAATA – 3‘
FOSL1 pre-mRNA (intron 3 – exon 4)	5‘ – CCTCAGAACCCTGAGTCCAA – 3‘
	5‘ – CTTCTGCTTCTGCAGCTCCT – 3‘

FOSL1 mRNA (exon 3 – exon 4)	5′ – CAGGCGGAGACTGACAAACT – 3′
	5′ – CTTCCAGCACCAGCTCTAGG – 3′

* Distance from transcription start site

Supplementary figure legend

Figure S1. TALE-HME expression and effects on chromatin

(A) Expression of the TALE-HME constructs was confirmed by Western blot using an anti-HA antibody against the HA-linker or GFP in case of TALE-GFP.

(B) The effect of H3K9me2 is specific, as tethering TALE-G9a did not result in increased levels of H3K9me3 at the target exon.

(C) TALE-ASH1L did not increase H3K36me3 levels at the target exon and had no effect on EDB inclusion as confirmed by ChIP (left) and RT-qPCR (right). ChIP signals are calculated as immunoprecipitated DNA over input relative to total H3 signal and normalized to an intergenic, non-transcribed region on chromosome 10. Amplicon positions within the *FN1* gene are indicated in the gene diagram at the bottom. EDB inclusion rates are calculated as the ratio of the EDB exon to the upstream constitutive exon 24 and normalized to TALE-GFP. Mean ± SEM are shown, n = 3-4. Statistical significance was analyzed by t-test.

Figure S2. H3K9me3 signal at internal exons

Relative H3K9me3 abundance profiles in K562 (top panels) and MCF-7 (bottom panels) cells 1kb up- and downstream of transcriptional start sites, poly-adenylation sites, 3′ and 5′ splice sites of internal exons were plotted versus relative genomic positions 1kb up- and downstream of gene architecture hallmark of interest. Genome wide abundance profiles were derived from chromatin immunoprecipitation sequencing (ChIP-seq) experiments performed in the ENCODE project. Schematic gene architecture hallmarks are displayed above.

Figure S3. Induction of HME by doxycycline in stable cell lines

A) Inducible expression and nuclear localization of JMJD2D-mRFP and Suv39H1-mRFP was confirmed by mRFP fluorescence.

B) Western blot showing the inducible expression of JMJD2D-mRFP and Suv39H1-mRFP. Stable cell lines were treated with 15 or 30 µg doxycycline resulting in a global decrease of H3K9me3 levels after JMJD2D induction and an increase in the case of Suv39H1.

C) Transient expression of TALE-HME constructs targeting *FOSL1* exon was confirmed by Western blot using an anti-HA antibody against the HA-linker or GFP in the case of TALE-GFP.

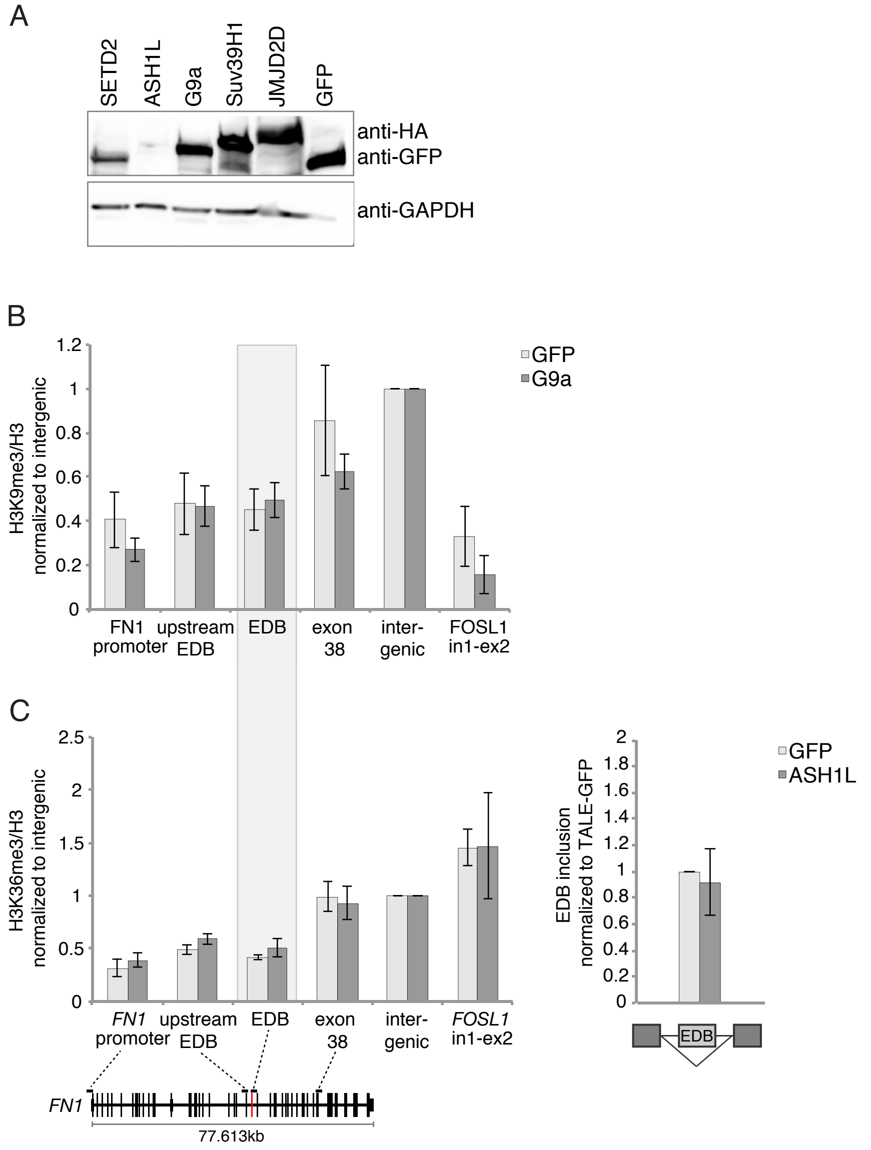
A
SETD2
ASH1L
G9a
Suv39H1
JMJD2D
GFP
anti-HA
anti-GFP
anti-GAPDH
B
H3K9me3/H3 normalized to intergenic
GFP
G9a
FN1 promoter
upstream EDB
EDB
exon 38
inter-genic
FOSL1 in1-ex2
C
H3K36me3/H3 normalized to intergenic
FN1 promoter
upstream EDB
EDB
exon 38
inter-genic
FOSL1 in1-ex2
FN1
77.613kb
EDB inclusion normalized to TALE-GFP
GFP
ASH1L
EDB

Figure S1

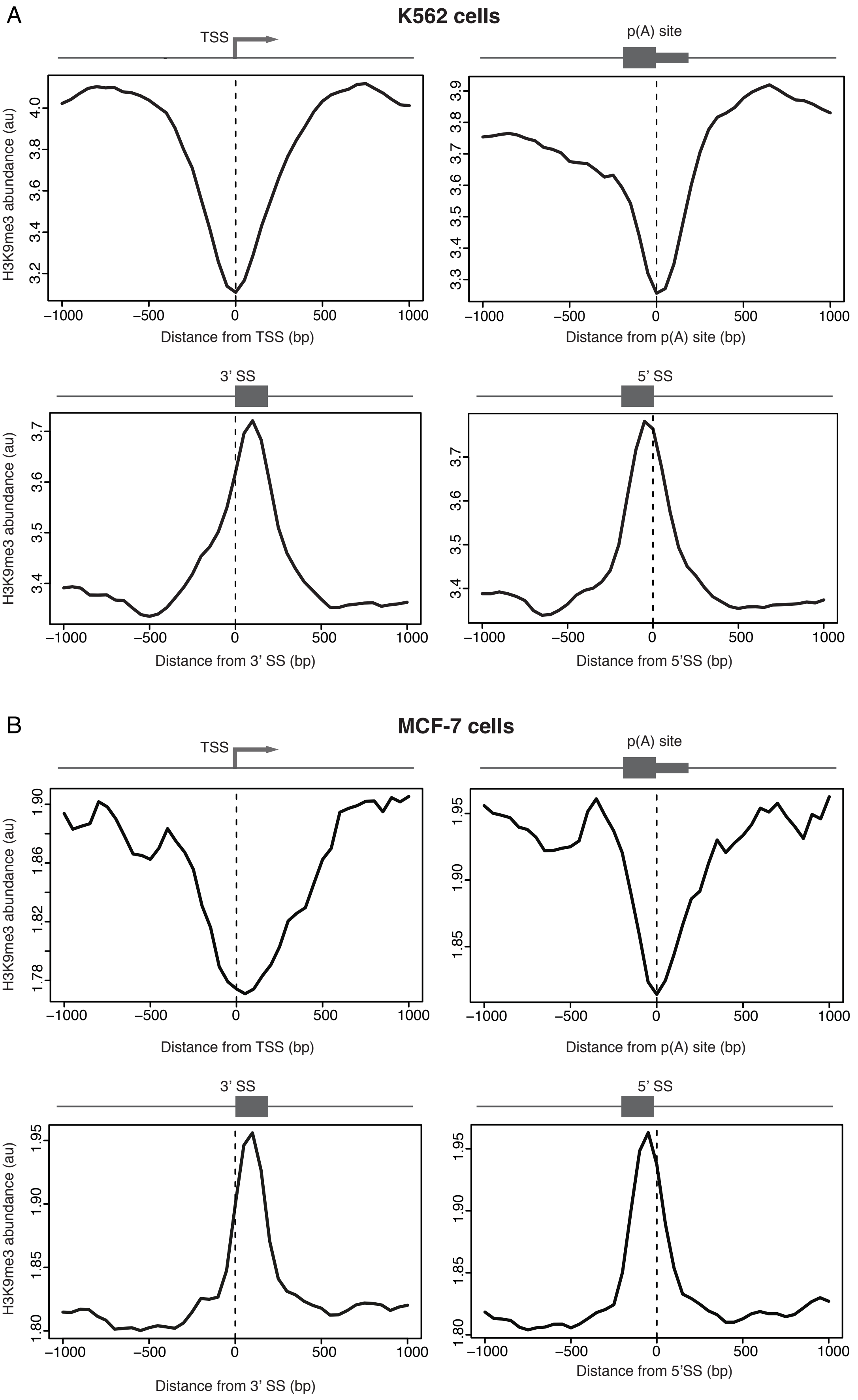
A
K562 cells
TSS
p(A) site
3' SS
5' SS
H3K9me3 abundance (au)
Distance from TSS (bp)
Distance from p(A) site (bp)
Distance from 3' SS (bp)
Distance from 5'SS (bp)
B
MCF-7 cells

Figure S2

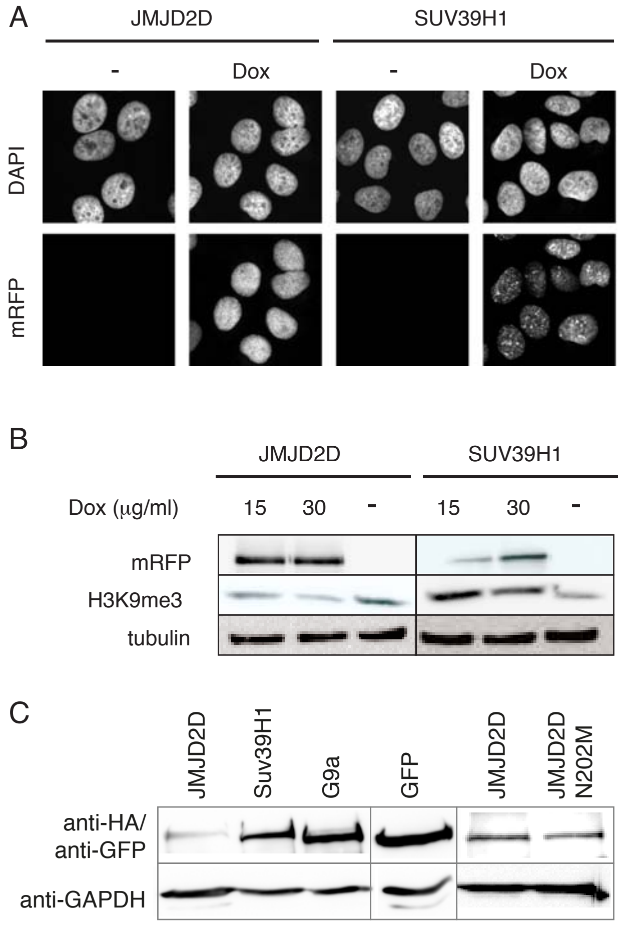
A
JMJD2D
SUV39H1
-
Dox
-
Dox
DAPI
mRFP
B
JMJD2D
SUV39H1
Dox (μg/ml)
15
30
-
15
30
-
mRFP
H3K9me3
tubulin
C
JMJD2D
Suv39H1
G9a
GFP
JMJD2D
JMJD2D N202M
anti-HA/
anti-GFP
anti-GAPDH
